# Phytoproduct, Arabic Gum and *Opophytum forsskalii* Seeds for Bio-Fabrication of Silver Nanoparticles: Antimicrobial and Cytotoxic Capabilities

**DOI:** 10.3390/nano11102573

**Published:** 2021-09-30

**Authors:** Kawther Aabed, Afrah E. Mohammed

**Affiliations:** Biology Department, College of Science, Princess Nourah Bint Abdulrahman University, Riyadh 84428, Saudi Arabia

**Keywords:** bio-nanotechnology, *Candida* sp., bacteria, LoVo cancer cell lines, biomolecules

## Abstract

The application of biological materials in synthesizing nanoparticles has become significant issue in nanotechnology. This research was designed to assess biogenic silver nanoparticles (AgNPs) fabricated using two aqueous extracts of *Acacia arabica* (Arabic Gum) (A-AgNPs) and *Opophytum forsskalii* (Samh) seed (O-AgNPs), which were used as reducing and capping agents in the NPs development, respectively. The current study is considered as the first report for AgNP preparation using *Opophytum forsskalii* extract. The dynamic light scattering, transmission electron microscopy, and scanning electron microscopy were employed to analyze the size and morphology of the biogenic AgNPs. Fourier transform infrared (FTIR) spectroscopy and chromatography/mass spectrometry (GC-MS) techniques were used to identify the possible phyto-components of plant extracts. The phyto-fabricated NPs were assessed for their antibacterial activity and also when combined with some antibiotics against *Staphylococcus aureus* (Gram-positive) and *Pseudomonas aeruginosa* and *Escherichia coli* (Gram-negative) and their anticandidal ability against *Candida albicans* using an agar well diffusion test. Furthermore, cytotoxicity against LoVo cancer cell lines was studied. The results demonstrated the capability of the investigated plant extracts to change Ag^+^ ions into spherical AgNPs with average size diameters of 91 nm for the prepared O-AgNPs and 75 nm for A-AgNPs. The phyto-fabricated AgNPs presented substantial antimicrobial capabilities with a zone diameter in the range of 10–29.3 mm. Synergistic effects against all tested strains were observed when the antibiotic and phyto-fabricated AgNPs were combined and assessed. The IC_50_ of the fabricated O-AgNPs against LoVo cancer cell lines was 28.32 μg/mL. Ten and four chemical components were identified in *Acacia arabica* (Arabic Gum) and *Opophytum forsskalii* seed extracts, respectively, by GC-MS that are expected as NPs reducing and capping agents. Current results could lead to options for further research, such as investigating the internal mechanism of AgNPs in bacteria, *Candida* spp., and LoVo cancer cell lines as well as identifying specific molecules with a substantial impact as metal-reducing agents and biological activities.

## 1. Introduction

The spread of infectious diseases brought about by different pathogenic microorganisms and the rise of antibiotic resistance has led researchers worldwide to study new antimicrobial agents. Nanotechnology has had significant scope in recent research to deal with the synthesis and use of particles that range from around 1–100 nm in size [1]. Under this size range, all features (physical, chemical, and biological) change for single atoms/molecules from their similar bulk composition. Nanoparticles are formed worldwide in high amounts for use in a wide range of applications, such as environmental, healthcare, biomedical, cosmetics, food, and drugs. Nanoparticles have developed quickly in several aspects due to their current or enhanced characteristics based on their size, morphology, and distribution [2,3]. Ag has a great history of anti-microbial use to control infections in microbes beginning with the Phoenicians who applied Ag as a natural biocide to coat milk vials. Ag is a known antimicrobial factor against a broad array of over 650 microorganisms from different kinds of fungi, viruses, and Gram-negative or Gram-positive bacteria. A medical method called Ayurveda from ancient India defined Ag as a curative agent for several illnesses [1]. Recently, Ag has been used in the form of silver nanoparticles (AgNPs). Significant research has been published to date regarding the biological syntheses of AgNPs using plants and microorganisms, including bacteria and fungi. However, the use of plant extracts is useful for more than microbes. This is also an excellent technique to synthesize nanoparticles as it provides a natural capping agent to stabilize AgNPs and is free from poisonous chemicals. Furthermore, the use of plant extracts reduces the cost of microorganism isolation and their growth media, which increases the competitive feasibility of nanoparticle synthesis via microorganisms. A large number of plants were tested for AgNPs synthesis, which has emerged in many publications, such as leaves extracts of *Rosa rugosa* [4], *Psidium guajava* [5], *Stevia rebaudiana* [6], Different *Olea Europaea* trees [7], *Nymphae odorata* [8], as well as *Salvia officinalis* [9]. These AgNPs have shown the potential for storage in membranes and can later access cells, which damages the cell membranes or walls. Other mechanisms have been proposed, such as the interactions of Ag molecules with vital macromolecules such as enzymes and DNA [10]. Gum from *A. arabica* and *O. forsskalii* seeds were the two plant sources used here as the bio-mediator for AgNP synthesis in the current study. *Acacia* is an important genus of the family Leguminosae, and it is presumed that there are approximately 1380 species of Acacia worldwide. This genus has spread in arid and semi-arid zones of Africa and Asia. Arabic gum Acacia and Senegal (*A. senegal*) also belong to the Leguminosae family. These are famous medicinal plants in the Arabian Peninsula and the Sudan region in Africa [11]. Moreover, these are called (Kher) in Rajasthan (India) and other countries [12]. The root, bark, leaves, flowers, and gum have been used to treat diarrhea, dysentery, diabetes, eczema, skin diseases, cough, and asthma. On the other hand, these are also a source of polyphenols. Phytochemicals include several groups, such as resins, oleosins, volatile essential oils, alkaloids, phenols, phenolic glycosides, steroids, terpenes, and tannins [13]. Recently, these were used as supplements in pharmaceuticals, foods, and other manufactures in the USA and Europe [14]. Additionally, Arabic gum has been approved by the US Food and Drug Administration as a supplementary food. *O. forsskalii* belongs to the Aizoaceae family, which is widely spread in Palestine, Egypt, Qatar, Bahrain, and is abundant in Saudi Arabia (KSA), particularly in the northeastern part of the Aljouf region (Al-Adare and Bassita regions). As it has the highest content of carbohydrates, protein, and fats, the people in KSA use the seeds of *O. forsskalii* as food and mix the powder with dates and butter to prepare a traditional recipe called Pakilla. Moreover, this wilderness plant has also noted medicinal effects and has been utilized by local populations. Despite its great value, the plant has been used in limited applications. Information regarding the phytochemical and characterization of different parts of *O. forsskalii* are lacking. Such research provides data on the biologically active part, which may permit suitable applications to treat several diseases. Here, we address a non-toxic and environmentally friendly (green chemical) process for the synthesis of AgNPs by reducing Ag^+^ ions using aqueous extracts of gum from *A. arabica* and dried seeds from *O. forsskalii*. To our knowledge, it is the first time that *O. forsskalii* seeds are used as a biomediator in AgNPs formation. The phyto-fabricated nanoparticles were identified via SEM, TEM, and hydrodynamic radius (size from DLS) and zeta potential analysis. Moreover, the antimicrobial activity was examined against *S. aureus* (Gram-positive), *P. aeruginosa* and *E. coli* (Gram-negative), and fungal (*C. albicans*) strains to determine the minimum inhibitory concentration (MIC) and tolerance level. The cytotoxic effect was also determined against LoVo cancer cell lines using a 3-(4,5-dimethyl-2-thiazolyl)-2,5-diphenyl-tetrazolium bromide (MTT) assay. There are no available reports regarding the usage of *O. forsskalii* seed powder as a reducing agent; therefore, this study is considered as a first regarding *O. forsskalii* for AgNP fabrication.

## 2. Material and Methods

### 2.1. Materials

Silver nitrate (AgNO_3_) was obtained from Saudi Overseas Marketing and Trading Company (SOMATCO), Riyadh, Saudi Arabia. The nutrient agar plates and nutrient broth (Difco, Becton, Dickinson and Company, Sparks Glencoe, MD, USA), as well as the potato-dextrose agar (PDA) plates (Difco, Becton, Dickinson and Company, Sparks Glencoe, MD, USA), were purchased from Wateen Alhaiah Company, Riyadh, Saudi Arabia. All clinical bacterial isolates were obtained from the Microbiology Laboratory of the Princess Nourah Bint Abdulrahman University, Riyadh, Saudi Arabia. Antibiotic discs were obtained from OXOID^TM^ United Kingdom at the following concentrations: bacitracin, 10 µg/mL; ciprofloxacin, 10 µg/mL; tetracycline, 30 µg/mL; cefixime, 5 µg/mL. Furthermore, plant identities were verified and taxonomically classified. The plant parts were cleaned with distilled water, air dried, and ground into a fine powder using a milling machine (IKA WERKE, GMBH, Staufen im Breisgau, Germany). The powder was stored at room temperature in plastic bags until use.

### 2.2. Preparation of Aqueous Extracts for AgNP Fabrication

#### 2.2.1. Extraction Method

The aqueous plant extracts were prepared by mixing 10 g of single-plant powder with 100 mL of distilled water. The mixture was heated for 10 min at 80 °C and then filtered through ‘Whatman filter paper Grade 1’ and further filtered through ‘Whatman filter paper Grade 3’ for additional purification and kept in the refrigerator for further use [15]. 

#### 2.2.2. Synthesis of AgNPs

To synthesize AgNPs, a volume of approximately 10 mL for each plant extract (10 mg/mL) was added to 90 mL of AgNO_3_ (1 mM) solution in a flask and kept under dark conditions to allow the reactions to progress at room temperature for 48 h.

### 2.3. Characterization of Biogenic AgNPs

Different methods for the characterization of biogenic AgNPs prepared here were used to provide additional characteristics besides the observed color change as the first sign for Ag ion conversion to AgNPs.

#### 2.3.1. Dynamic Light Scattering (DLS) and Zeta Potential

The size distribution patterns were evaluated using a DLS technique, and knowledge of the electrical charge of particles through the zeta potential was measured with a Zetasizer (Nano ZSP, Malvern Instruments Ltd., version 7.11, Malvern, UK). NPs were dissolved in 1 mL water and placed in the cuvette for the measurements.

#### 2.3.2. Transmission Electron Microscopy (TEM) and Scanning Electron Microscope (SEM)

The particle size distribution and morphology of the biogenic AgNPs were investigated via TEM (JEM-1011, JEOL, Tokyo, Japan) using an acceleration voltage of 80 kV. All samples were prepared by drop-coating on carbon-coated (200 mesh) TEM grids. Furthermore, SEM (JEOL, JED-2200 series, Tokyo, Japan) was used for surface analysis of NPs. 

#### 2.3.3. Fourier Transform Infrared (FTIR) Spectroscopy

The FTIR measurements were performed to identify the potential biomolecules in the plant extract responsible for reducing and capping the AgNPs. The spectra were recorded using FTIR spectroscopy (Spectrum100, Perkin-Elmer, Wellesley, MA, USA,) with a diffuse reflectance accessory, and the scanning data were obtained in the range of 450–3500 cm^−1^.

### 2.4. Antibacterial Susceptibility Testing (AST)

The antibacterial activity of AgNPs was evaluated against three pathogenic bacteria, *S. aureus* (Gram-positive), *P. aeruginosa* and *E. coli* (Gram-negative), and a fungal strain (*C. albicans*) using an agar well diffusion assay. Pure cultures for each bacterial strain were sub-cultured on nutrient agar plates and grown for 24 h at 37 °C. The direct colony suspension method using a McFarlane 0.5 bacterial suspension (1.5 × 10^8^ CFU/mL) in a saline tube was prepared using the McFarland standard. The fungal strain was sub-cultured in potato dextrose agar and incubated for 24 h at 25 °C. Test plates were inoculated using testing strains with sterile swabs. Thereafter, three wells in each plate contain tested microbes prepared and filled with biogenic AgNPs. Sterile distilled water was used as the negative control, and antibiotic discs were used as the positive controls. One hour later, the treatment plates were inverted and incubated at 37 and 25 °C for the bacterial strain and *C. albicans*, respectively, for 24 h. The zone of inhibition (mm) was measured around the well and antibiotic discs.

#### 2.4.1. Minimum Inhibitory Concentration (MIC) and Minimum Bactericidal Concentration (MBC)

A microdilution method using a nutrient broth (NB) medium was applied to detect the MIC and MBC. 10 mL of 5 × 10^8^ CFU/mL for each investigated bacteria was introduced individually into 10 mL of NB. Thereafter, the bacterial tubes were incubated following the addition of AgNPs in different concentrations for 24 h. The MICs were determined by testing the bacterial growth using turbidity as an indicator and is considered as the concentration that suppressed 99% of the bacterial. The MBC is known as the lowest concentration that displayed growth [16].

#### 2.4.2. Tolerance Level 

The tolerance level may help determine differences between the bactericidal and bacteriostatic abilities for AgNPs against the tested microbes. The MBC:MIC ratios were calculated where bacteriostatic agents were considered for MBC:MIC ≥ 4, and agents were bactericidal for those ≤ 2 [17,18].

### 2.5. Synergistic Effect of AgNPs and Antibiotics

The disk diffusion method was used to test the effect of antibiotics with AgNPs against the tested microbes. An AgNPs concentration of 20 μg was added to the antibiotic discs Tetracycline, Bacitracin, Ciprofloxacin, Cefixime, Fluconazole, and Metronidazole. The antibiotic discs without any additions were used as controls. The treated plates were incubated for 24 h at 37 °C, and the inhibition zone was determined.

### 2.6. Field Emission Scanning Electron Microscope ((FESEM)

Hitachi S-4500 FESEM (Schaumburg, IL, USA) was employed to detect morphological alterations of NPs-treated microorganisms. A drop from treated microorganisms was placed in grids from carbon-coated copper then dried by exposing for 5 min to a mercury lamp. 

### 2.7. Cytotoxicity of AgNPs

#### Cell Lines and Culture

Human colon carcinoma (Lovo) cell lines were tested in KSU, Riyadh, Saudi Arabia. Cells were attained from the Leibniz Institute DSMZ-German Collection of Microorganisms and Cell Cultures (Braunschweig, Germany). The cells were kept in a high-glucose Dulbecco’s Modified Eagle’s Medium (DMEM) with 10% serum of fetal bovine (Gibco, Darmstadt, Germany) and 1% penicillin-streptomycin solution (Thermo, Mississauga, Ontario, Canada) at 37 °C and a CO_2_ concentration of 5% in a humidified incubator. Approximately 5 × 10^5^ cells were cultured in 24-well plates overnight (NEST, Wuxi, China). Biogenic AgNPs were applied at different concentrations inside the cultures and the negative control was the vehicle for the tested cells. After 2 days, cells in 100 mL of (5 mg/mL) 4,5-dimethylthiazol-2-yl-2,5-diphenyltetrazolium bromide (MTT) were incubated for 2 h at 37 °C. The absorbances were measured at 595 nm with an ELISA reader and the cell viability was calculated using the formula:

Cell viability 100% = (OD Sample/OD Control) × 100.

Cytotoxicity% = 100 − Viability%

The half-maximum inhibitory concentration (IC_50_) values represent the concentration of biogenic AgNPs required for 50% inhibition of cell growth.

### 2.8. Chromatography/Mass Spectrometry (GC-MS) Techniques

The chemical analyses of *Acacia arabica* (Arabic Gum) and *Opophytum forsskalii* (Samh) seed were tested via GC-MS (Agilent Technologies 220 Ion Trap GC/MS, Santa Clara, CA, USA). Helium was applied as the carrier gas using a column with a flow rate of 1 mL/min; pressure of 8.2317 psi; average velocity of 36.623 cm/s; holdup flow of 1.3653 min; post run of 0.99996 mL/min; column maximum temperature of 450 °C; and sized at 30 m × 250 µm × 0.25 µm). The original temperature was 70 °C for 52 min before reaching 250 °C. The chemical components were identified from the National Institute of Standards and Technology (NIST) chemical database.

### 2.9. Statistical Analysis

Ag NPs images were chosen for characterization from one of the triplicates. GraphPad PRISM 9.1 (San Deigo, CA, USA) was used to calculate the means ± standard deviations and to produce the cytotoxicity level and graph preparation. 

## 3. Results and Discussion

This investigation was designed to test the ability of the two biogenic materials *A. arabic* gum (A) and *O. forsskalii* seeds (O) in AgNP fabrication and to check the A-AgNPs and O-AgNPs activities by testing their efficacy against some microbes and one specific cell line. The color of the reaction substrate gradually changed in a time-dependent manner when AgNO_3_ (colorless) was combined with each plant extracts separately providing a yellow color combination. Such conversion is known as the main indicator for the biotransformation of Ag^+^ ions into spherical Ag^0^ [19]. Many studies have reported color change as the primary indicator for the conversion of Ag^+^ ions to AgNPs [20,21,22], which is related to the excitation of the surface plasmon resonance (SPR). Usha and Rachel [23] indicated the AgNO_3_ conversion to brown when they added *A. nilotica* leaf extract for AgNP synthesis. Brown mixtures signal the typical excitation of surface plasmon vibrations in AgNPs [24]. Varied NPs colors indicating varied NPs characteristics such as size, shape, and stability [19]. 

**Figure 1:**
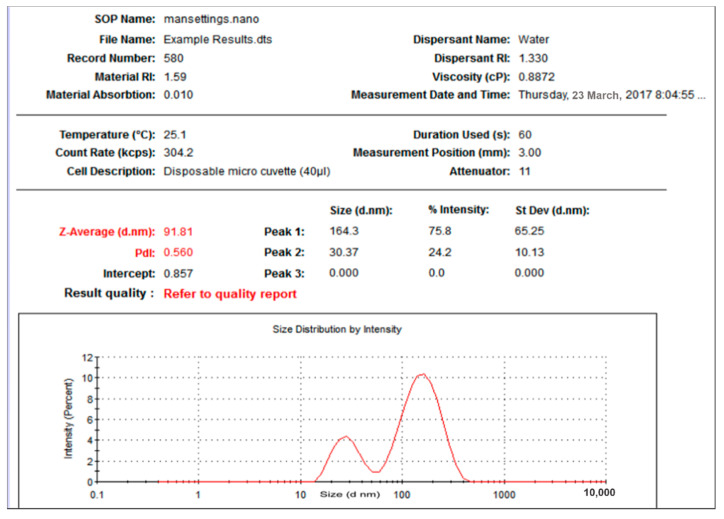
Size distribution of AgNPs capped with the biomolecules from *O. forsskalii*.

**Figure 2:**
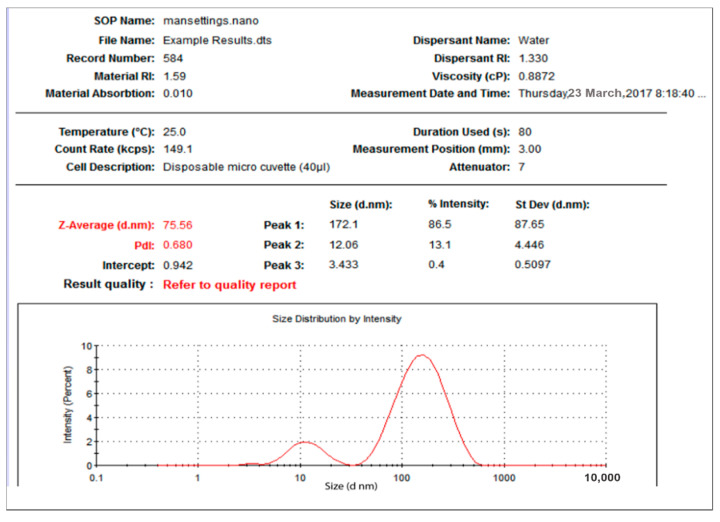
Size distribution of AgNPs capped with the biomolecules from *A. arabica* gum.

Further characterization was reported for DLS measurements for hydrodynamic radius (Figure 1 and Figure 2) indicating an average size of 91 nm for the prepared O-AgNPs and 75 nm for A-AgNPs. The particles’ sizes from intensity distribution are actually bimodal distributions with major particles having sizes of 164 nm (76%) and 172 nm (87%) for O-AgNPs and A-AgNPs, respectively. Figures indicating size distribution of the three reading for each NPs are attached as Figure S1 and S2 for O-AgNPs and A-AgNPs, respectively.

The zeta potentials were −0.34 and −0.32 for the prepared O-AgNPs and A-AgNPs, respectively, showing a negative charge for both NPs. High negative zeta potential values may lead to higher AgNP stability as this gives a high repulsion without particle accumulation [25]. However, low negative zeta potential values detected here could result in fast NPs agglomeration. Recent studies have indicated negative values of phyto-fabricated AgNPs [22,26,27,28] when investigating different plants. Negative charges may be achieved from phytochemicals adhering to Ag ions as negative groups [29]. It is well known that phytochemicals in the extraction medium can reduce and stabilize AgNPs prepared by such biological means [30]. Consequently, different phytochemicals could be the main reason for different sizes and potentials in phyto-fabricated AgNPs. 

**Figure 3:**
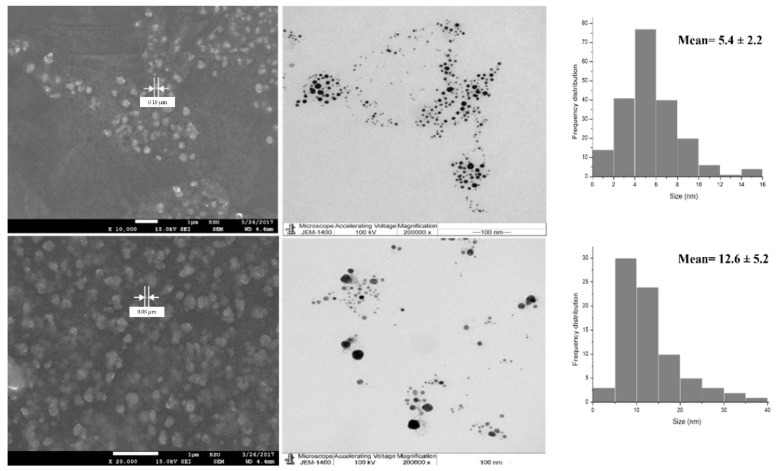
SEM image (**left side**) and the corresponding TEM image (**right side**) of AgNPs capped with the biomolecules from *O. forsskalii*. Scale bar represents 1 μm and 100 nm and magnification of 10,000, 20,000 and 200,000 for SEM and TEM images, respectively. Size measurements were analyzed by ImageJ software constructed from TEM micrographs.

**Figure 4:**
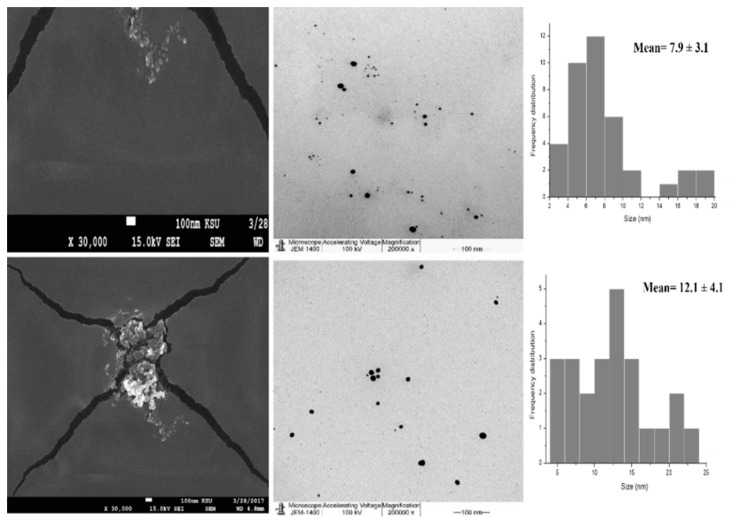
SEM image (**left side**) and the corresponding TEM image (**right side**) of AgNPs capped with the biomolecules from *Acacia arabica* gum Scale bar represents 100 nm and magnification of 30,000 and 200,000 for SEM and TEM images, respectively. Size measurements were analyzed by ImageJ software constructed from TEM micrographs.

Here, SEM and TEM images (Figure 3 and Figure 4) showed spherical shapes with no aggregated particles. Mean diameters of 5.4 and 12.6 nm for O-AgNPs and 7.9 and 12.1 nm for A-AgNPs were observed via TEM image analysis using ImageJ software, which agrees with the size detected using DLS measurements (hydrodynamic radius). Additional images regarding NPs distribution and size analysis from TEM images are added as Supplementary Materials (Figure S3 and S4) for O-AgNPs and A-AgNPs, respectively. Variation between particle size using both techniques is generally related to varied TEM and DSL measurements principles. A size of 20 nm and a spherical shape were also detected by Saratale et al. [31] using SEM for AgNPs fabricated using *A. nilotica* leaf extract. The EM results showed good distributions for both phyto-fabricated AgNPs. 

**Figure 5:**
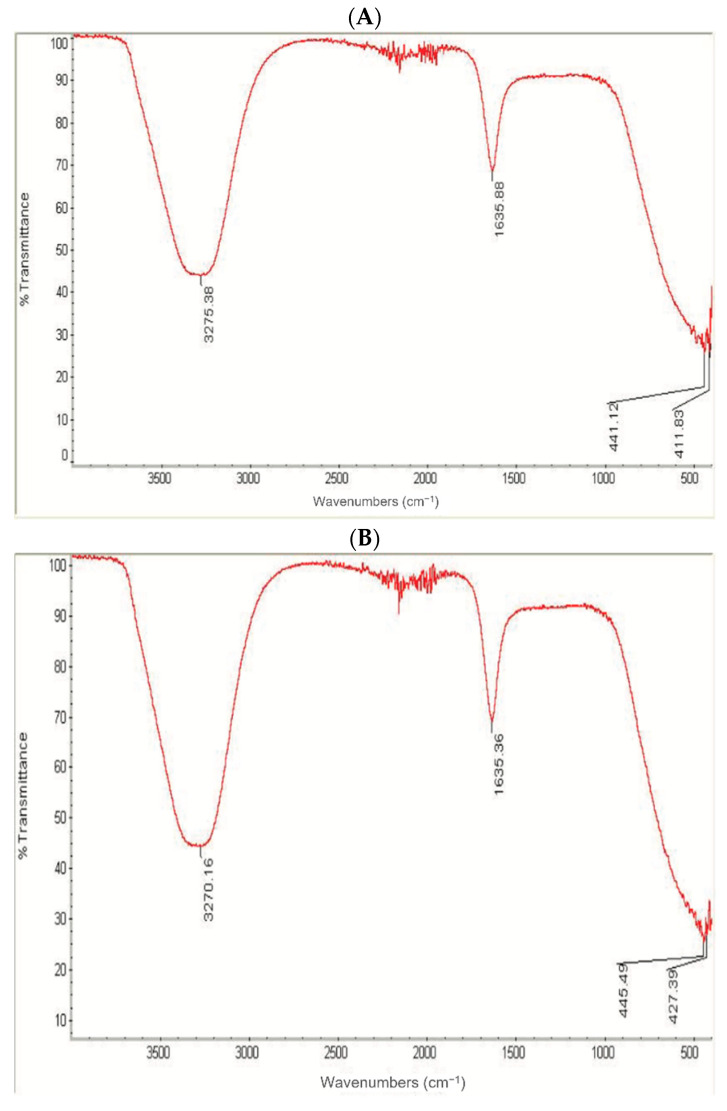
FT-IR images of AgNPs obtained using *O. forsskalii* seeds (**A**) and for those prepared by *A. arabica* gum (**B**).

FTIR spectroscopy is an analytical method to identify organic and inorganic materials with the ability to acquire an absorption spectrum for solids, liquids, and gases [32]. Values of 3275 and 3270 cm^−1^ were detected for the spectrum of the fabricated A-AgNPs and O-AgNPs, respectively, (Figure 5) indicating the presence of the polyphenolic −OH group and N-H stretching of the amine as the detected values were approximately 3300 cm^−1^ [33]. Further absorption at 1635 cm^−1^ linked to amide I and carbonyl stretching proteins (C=O) as indicated by Leela and Anchana [32] and Khandel et al. [34] was observed for AgNPs fabricated from both plant types. The stability of proteins even after binding with AgNPs is expected as 1635 cm^−1^ was documented for natural protein [35]. The presence of different compounds such as phenol and protein detected by various peaks show their role in fabricating AgNPs as well as stabilizing and capping molecules. Some metabolites are involved in the biofabrication of AgNPs, such as terpenoids, saponins, flavonoids, and quinines [36]. *A. nilotica*, which is known as *A. arabica* gum, is comprised of alkaloids, volatile essential oils, and phenols, such as saponins as well as flavonoids, flavonols, phenolics, and high proteins were also noted in *O. forsskalii* seeds [13,37]. Such chemical constituents could be the main factors that help when using *A. arabica* gum and *O. forsskalii* seeds for AgNP fabrication. Two phases are expected to be involved in the bioreduction of AgNPs. These are: (1) aqueous solution that contains positively charged ions adhered to biological materials that contain negatively charged biomolecules, and (2) secretion of some cellular enzymes that help in the conversion of metal-to-metal nanoparticles [38]. The results reveal the ability of *A. arabica* gum and *O. forsskalii* seeds to fabricate AgNPs and give small-sized particles. This is beneficial in different nanomaterial applications that encourage the phyto-fabrication of NPs.

### 3.1. Antimicrobial Capability of Phyto-Fabricated AgNPs

**Figure 6:**
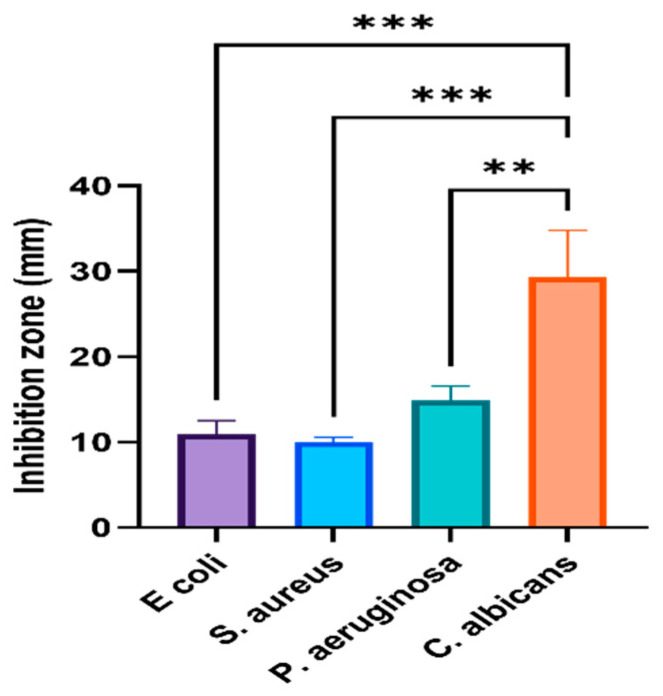
Activity of AgNPs fabricated by *O. forsskalii* seeds. One-way ANOVA with was performed to identify differences between groups. *p* < 0.001 (***), *p* < 0.01 (**).

**Figure 7:**
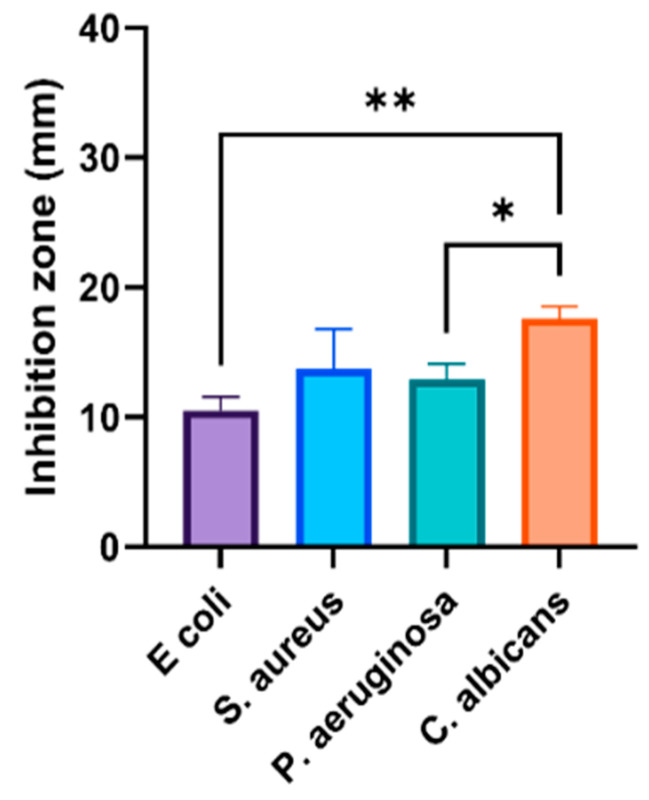
Activity of AgNPs fabricated by *A. arabica* gum. One-way ANOVA with was performed to identify differences between groups. *p* < 0.01 (**), *p* < 0.05 (*).

Finding alternatives to control the spread of antibiotic-resistant microorganisms is an urgent issue. Phyto-fabricated AgNPs with the aid of plant extracts could be an option as a high bactericidal ability against the tested microbes was reported here. A higher antibacterial ability was observed for phyto-fabricated AgNPs compared with AgNO_3_ and no activity was observed for plant extracts alone, indicating that 10 mg/mL might not be sufficient for antibacterial activity. However, Sadiq et al. [39] exhibited an antibacterial ability against *E. coli* and *Salmonella* sp. as treated with *A. nilotica* (leaves, pods, and bark). Currently, the phyto-fabricated O-AgNPs showed an antibacterial ability against the tested bacterial strains with the highest activity for *Pseudomonas* sp. followed by *E. coli* and then *S. aureus* Significant variations were noted (*p* < 0.001 and *p* < 0.0001) (Figure 6). This trend suggests that the low ability was observed against Gram-positive bacteria due to the difficulties of AgNPs to enter cells, which could be a consequence of the bacterial wall structure and the thick layer of peptidoglycan [40] as well as the easy entry of AgNPs into Gram-negative bacteria [41]. However, no special trend was detected for the prepared A-AgNPs, where the highest ability was observed against *S. aureus*, which was followed by *Pseudomonas* sp. and *E. coli* (Figure 7).

**Figure 8:**
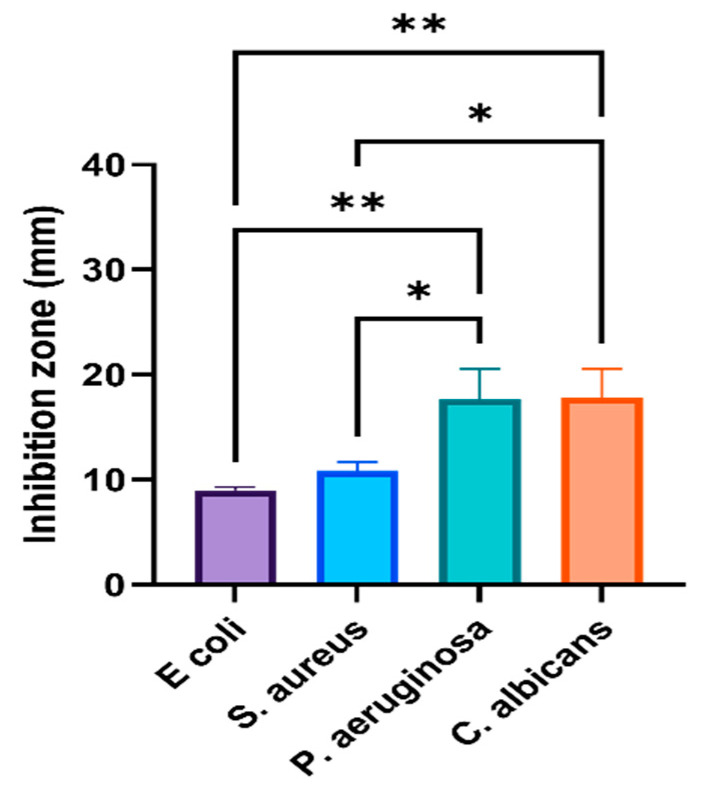
Activity of AgNO_3_ against tested microbes. One-way ANOVA with was performed to identify differences between groups. *p* < 0.01 (**), *p* < 0.05 (*).

Some studies have indicated a positive relationship between the high activity of biogenic AgNPs and Gram-negative bacteria [21,42], but others did not find a special pattern nor a link between the cell wall structure and biogenic AgNPs activity [43]. This could be related to the fact that AgNPs are bacteria-specific or that their efficiency is related to special biomolecules that differ based on the plant source. Interestingly, a higher anticandidal ability was observed against *C. albicans* for AgNPs fabricated by both plant materials compared to their activity against bacterial strains. However, the prepared O-AgNPs showed a significantly higher anticandidal effect on *C. albicans* (29 ± 2.5 mm) compared with A-AgNPs (17 ± 0.9 mm). A recent study that used AgNPs fabricated with the seeds of *Syzygium cumini* found a strong suppression of *Candida* spp. growth and enzyme production ability [22]. The phyto-fabricated A-AgNPs showed higher activity against all tested microbes with significant variations (*p* < 0.001 and *p* < 0.0001) (Figure 7) compared to plant extract and Ag^+^ ions (Figure 8), which may be a consequence of their combined activities. On the other hand, AgNPs generally have a large surface area because of the small particle size and can easily attach and penetrate bacterial cell walls [41]. This could be the main reason for their higher activity in relation to Ag ions against the tested microbes. Similar effect patterns of A-AgNPs and O-AgNPs prepared here against microbes could be because there were no significant variations in the characteristics AgNPs from both plant types. Furthermore, approximately 69% and 96% of Bacitracin and Cefixime activities against *S. aureus* were detected. More than 70% of Tetracycline activity against *Pseudomonas* sp. was noted, and about 63% of Fluconazole and double the Metronidazole activity were noted against *C. albicans*. Compared to the antibiotic effect, phyto-fabricated O-AgNPs displayed 94% of Tetracycline activity against *E. coli*, more than 100% of Bacitracin activity, and 42% of Cefixime activity.

Furthermore, phyto-fabricated A-AgNPs showed 90% and more than 100% of Tetracycline and Bacitracin activities, respectively, against *E. coli*. More than 50% of Tetracycline and Ciprofloxacin activities were also noted. About 96% and more than 100% of Bacitracin and Cefixime activities against *S. aureus* were reported, and about 61% of Tetracycline activity against *Pseudomonas* sp. was detected. For fungicides, 36% of Fluconazole and more than 100% of Metronidazole against *C. albicans* were observed for A-AgNPs. The phyto-fabricated AgNPs exhibited both MIC and MBC at their original concentration. For the tolerance level, this investigation is inconsistent with the results of Algebaly et al., [44] who revealed that the tolerance level is considered bactericidal for phyto-fabricated AgNPs by some plant extracts.

### 3.2. Synergistic Effect of Phyto-Fabricated AgNPs

**Figure 9:**
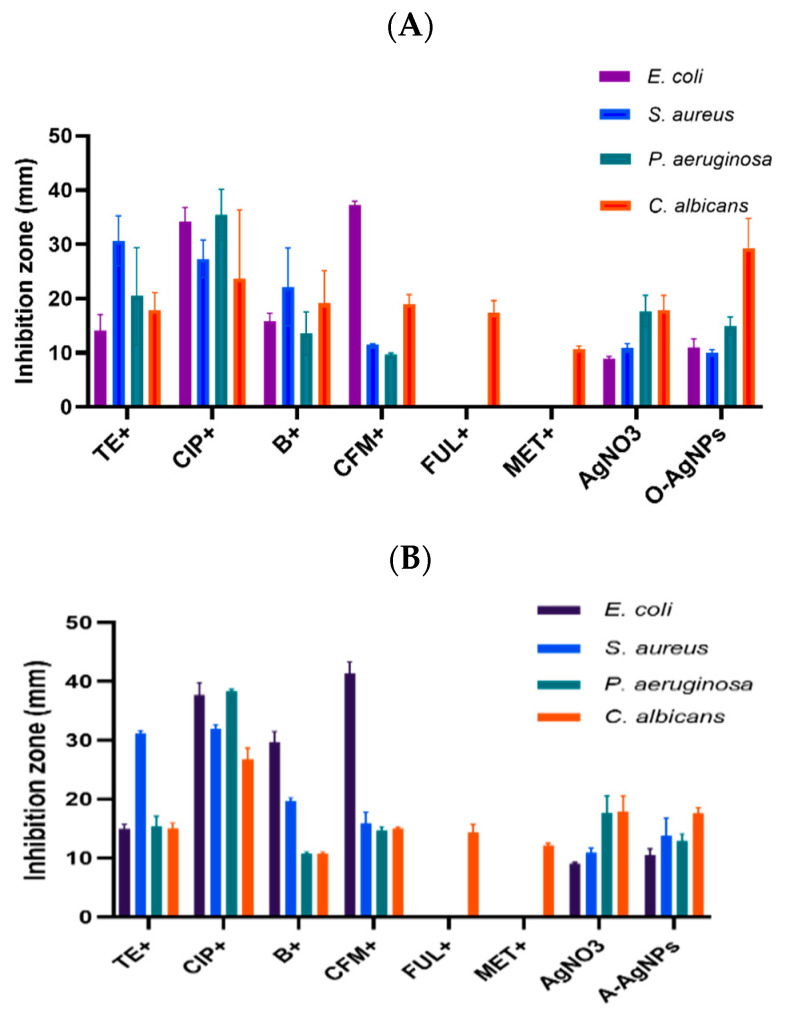
Synergistic effect of the antibiotics and AgNPs fabricated using *O. forsskalii* seeds (**A**) and synergistic effect of the antibiotics and AgNPs fabricated using *A. arabica* gum (**B**).

**Figure 10:**
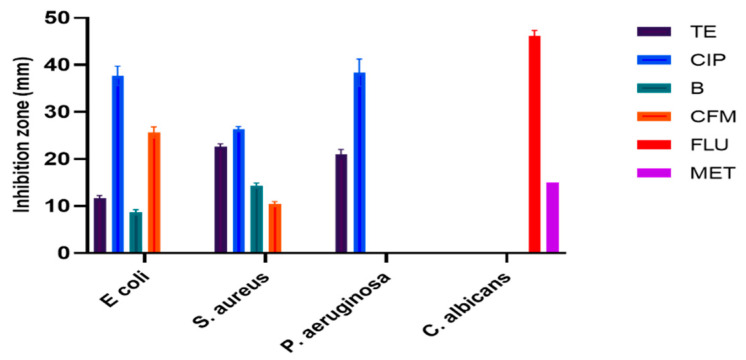
Activity of antibiotics against tested microbes.

The potentials of phyto-fabricated AgNPs and antibiotics were examined after their combination (Figure 9A,B). Higher antibacterial activities against all tested strains were observed for combinations compared with the antibiotic (Figure 10) and for phyto-fabricated AgNPs alone. Of note, the antibiotics (Tetracycline, Bacitracin, Ciprofloxacin, and Cefixime) showed no activity against *C. albicans*. However, when AgNPs were added to the antibiotic discs and examined, high antifungal activities were observed, which were even higher than that for the fabricated A-AgNPs alone. For *E. coli*, the maximum potential was observed with the synergistic effect of Cefixime, which is associated with O-AgNPs and A-AgNPs and caused inhibitions of 37.6 and 41 mm, respectively. The activities of Tetracycline, when conjugated to A-AgNPs and O-AgNPs, were 37.8 and 34.2 mm, respectively. After the association of Cefixime with AgNPs, its activity increased significantly from 25.6 to 37.6 mm when associated with O-AgNPs and to 41.4 mm when mixed with A-AgNPs. The same was observed for Bacitracin when associated with AgNPs as its efficacy increased significantly from 8.7 to 15.8 and 29.7 mm for O-AgNPs and A-AgNPs, respectively, when tested against *E. coli*. The highest activities were observed for Tetracycline and Ciprofloxacin against *S. aureus* when each was associated with AgNPs. The inhibition ability for Tetracycline increased from 22.6 to 29.7 and 31.1 mm and for Ciprofloxacin increased from 26.3 to 29 and 31.8 mm when associated with O-AgNPs and A-AgNPs, respectively. For *Pseudomonas*, the highest activity was observed for Ciprofloxacin (38.3 mm) and no change in the activity was observed when associated with either AgNPs type. Cefixime and Bacitracin showed no activity against *Pseudomonas*; however, after their association with AgNPs, their activities increased significantly and reached 19 and 22 mm when associated with O-AgNPs and to 15 and 10.6 mm when associated with A-AgNPs. For *C. albicans* the highest activity was noted for Ciprofloxacin when associated with O-AgNPs (26.8 mm). An antagonistic effect was observed when the fungicides Fluconazole and Metronidazole were associated with the phyto-fabricated AgNPs. The activity of Fluconazole was reduced when associated with A-AgNPs and O-AgNPs from 46.2 to 14.4 and 10.7 mm, respectively. This suggests that the phyto-fabricated AgNPs may suppress the chemical components in antibiotic discs. The combined activity of the AgNPs and antibiotics was in agreement with previous results for AgNPs using biogenic extracts [45,46,47]. Of note, antibacterial action for the antibiotic-associated AgNPs could be efficient for treating antibiotic-resistant bacteria in humans [21].

### 3.3. Morphological Studies on Treated Bacteria

**Figure 11:**
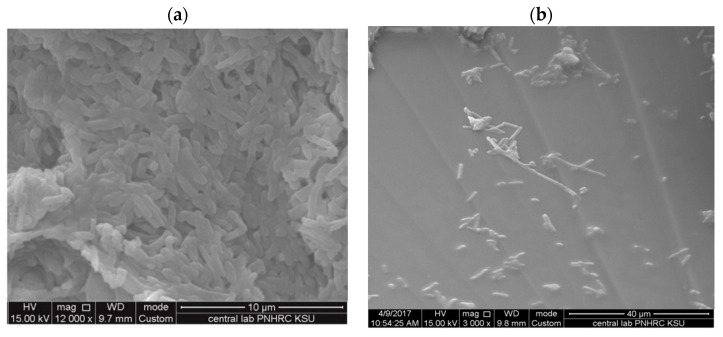
Morphology of untreated *Pseudomonas* sp. (**a**) and the morphological alteration (elongation) after application of O-AgNPs fabricated using *O. forsskalii* seeds (**b**).

**Figure 12:**
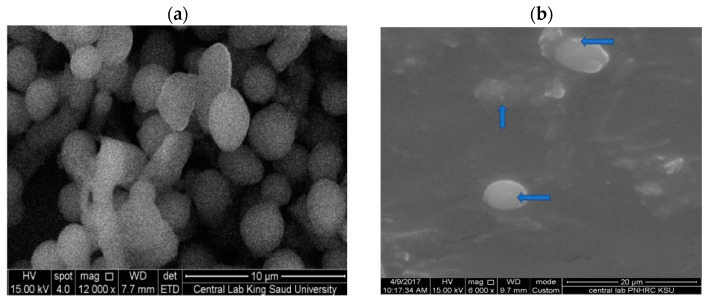
Morphology of untreated *C. albicans* (**a**) and the morphological changes after application of AgNPs fabricated using *O. forsskalii* seeds (**b**). The arrows show the AgNPs in the cell surface.

*P. aeruginosa* was subjected to phyto-fabricated O-AgNPs and then detected after 2 h under SEM to study any differences in the bacterial morphology to indicate the mechanism of AgNPs against microbes. Changes in the morphology and bacterial cell elongation were observed for *Pseudomonas* sp. (Figure 11), a similar trend of observation was also reported [21,48]. On the other hand, *C. albicans* was examined under SEM following the fabricated O-AgNPs treatment (Figure 12). An abnormal shape was observed and some AgNPs were noted on the cell surface. The accumulation and penetration of biogenic AgNPs inside *C. albicans* were also detected using TEM in addition to cell wall damage [22]. The mechanisms of AgNPs as antibacterial and anticandidal are not completely recognized. It is reported that the bactericidal ability of AgNPs could be linked to Ag^+^ [49,50] and with its binding ability with cell DNA and cytoplasm for leakage out of the cell [51]. It was reported that AgNPs can form pits and holes on cells, which damages the *C. albicans* membrane and causes cell death [52]. The reason for cell death was postulated also as the production of reactive oxygen species [53]. 

### 3.4. Cytotoxic Potential 

**Figure 13:**
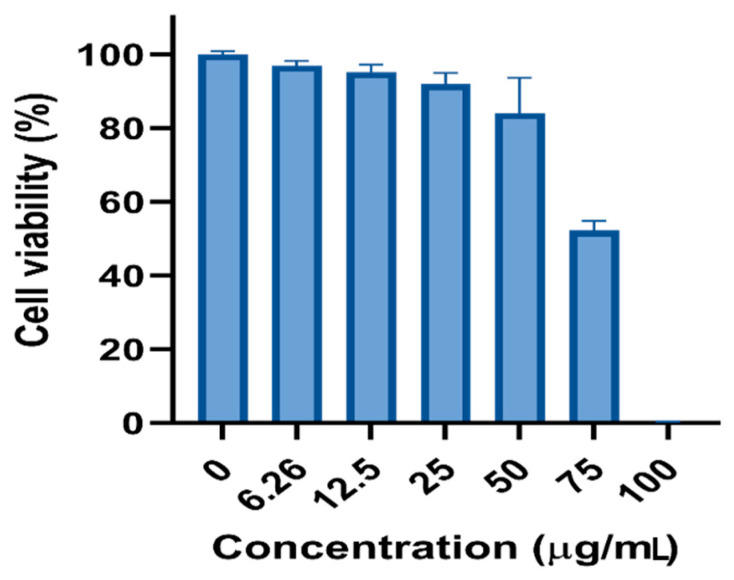
Effect of O-AgNPs on the viability of LoVo cancer cell lines. Bar graph showing dose relationship of O-AgNPs and the cell viability (%).

**Figure 14:**
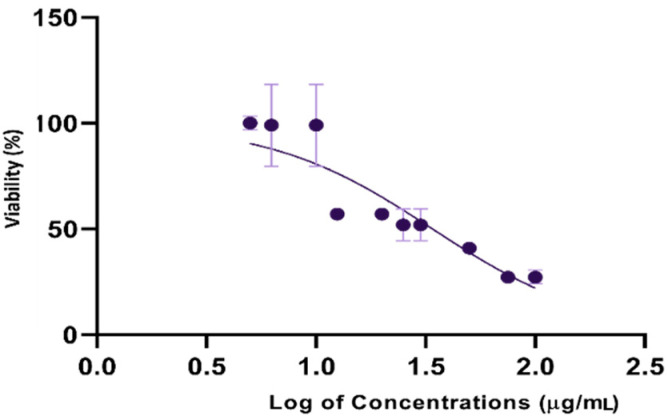
Log viability vs. normalized response-variable slope (three-parameter) of O-AgNPs on the normalized viability of LoVo cancer cell lines.

The suppression of cancer cells by chemotherapeutic agents is an efficient treatment, although the negative side effects are difficult to tolerate. Therefore, looking for alternatives without negative impacts is necessary. The phyto-fabricated AgNPs were tested against LoVo cancer cell lines by the MTT test. The results indicate that O-AgNPs had a cytotoxic ability against LoVo cancer cell lines in a dose-dependent reaction style. As the AgNPs concentration increased, the cytotoxic ability also increased in the order of 100 > 75 > 50 > 25 > 12.5 > 6.25 μg/mL (Figure 13). The IC_50_ (half-maximal) AgNP effect was determined after 48 h of treatments. The IC_50_ of the fabricated O-AgNPs against the LoVo cancer cell lines was 28.32 μg/mL (Figure 14). Some recent studies indicated the cytotoxic effect of biogenic AgNPs against LoVo cancer cells [43,54]; however, the A-AgNPs showed no effect. Although the entry of phagocytosis or endocytosis NPs to mammalian cells is largely size-dependent, [55]. Variations in cytotoxicity between NPs prepared by both plant origins were evident which could be related to the phyto-molecules capping each type. The cytotoxic activity of NPs is strongly related to free radical production, which leads to cell damage by attacking proteins and causes functionality losses with ultimately cell death [56,57,58]. The cytotoxic effect of AgNPs on LoVo cancer cell lines was mostly dependent on the particle size and dose [54]. The AgNPs showed an advantage as a promising candidate against LoVo cancer cell lines; however, the safe dose for living organisms must be determined as they have a high toxicity [59]. 

Furthermore, the chemical analysis of *Acacia arabica* (Arabic Gum) and *Opophytum forsskalii* (Samh) seed were examined via GC-MS analysis, and the active constituents are given in Table 1. The results indicate that both plants have 2,5-cyclohexadiene-1,4-dione,2,6-bis (1,1-dimethylethyl)- and Carboxin, Carbathiin; however, other components were also noted. Various phytochemical variants could be bio-reducing agents that helped to convert Ag ions into AgNPs and could be responsible for the enhanced biological activity of the prepared AgNPs. Such compounds were Prednisone and Hydrocortisone Acetate from the *Opophytum forsskalii* extract and 3,5-cyclohexadiene-1,2-dione,3,5-bis (1,1-dimethylethyl)-, phenol,2,4-bis (1,1-dimethylethyl)-, pentadecane, eicosane, 9,10-anthracenedione, 2-ethyl-, 9-octadecenoic acid (z)-, methyl ester, phenol, 4-(2-aminoproyl)-, and hydrocortisone acetate from the Arabic Gum extract.

## 4. Conclusions

Recently, there has been a growing concern among scientists worldwide to develop feasible alternatives for chemical medicinal compounds to resolve constraints, such as the microbial resistance to antibiotics and cancer drugs. The current investigation employed two medicinal plants *Acacia arabica* and *Opophytum forsskalii* for AgNPs phyto-fabrication as a simple method using their extracts, which act as reducing and capping agents for NPs. Nano-sized particles were obtained from both biogenic agents tested and a strong relationship between the phyto-fabricated AgNPs and the suppression of microbial and cancer cell growth and development was observed. Furthermore, phyto-fabricated AgNPs showed synergetic bactericidal and anticandidal abilities when associated with antibiotics as well as a cytotoxic ability against LoVo cancer cell lines was also noted. It is significant to indicate that the current study is the first report producing NPs from the *Opophytum forsskalii* seeds and investigated their biological activities. Slight variations in NPs behavior prepared from both plant materials could be related basically to the different biomolecules noted from each extract since no significant variations were noted for the NPs characteristics. Such molecules could be also varied in their ability to cap the NPs and in turn affect their biological activities. Biomolecules detected via FTIR and GC-MS were of interest to be separated and tested for their biological activities. Further investigations to determine the mode of action for phyto-fabricated AgNPs for applications in the biomedical field are required.

## Figures and Tables

**Table 1:** GC-MS analysis results of *Opophytum forsskalii* and arabic Gum extracts.

Compounds Identified in *Opophytum forsskalii* Seed Extract	Formula	Molecular Weight	R Time (min)
2,5-Cyclohexadiene-1,4-dione,2,6-bis (1,1-dimethylethyl)-	C_14_H_20_O_2_	220	66.573
Carboxin, carbathiin	C_12_H_13_NO_2_S	235	70.715
Prednisone	C_21_H_26_O_5_	358	115.640
Hydrocortisone acetate	C_23_H_32_O_6_	404	116.292
Compounds Identified in Arabic gum extract			
3,5-cyclohexadiene-1,2-dione,3,5-bis (1,1-dimethylethyl)-	C_14_H_20_O_2_	220	45.076
Phenol,2,4-bis (1,1-dimethylethyl)-	C_14_H_22_O	206	45.491
Pentadecane	C_15_H_32_	212	51.166
Eicosane	C20H42	282	51.166
2,5-Cyclohexadiene-1,4-dione,2,6-bis (1,1-dimethylethyl)-	C_14_H_20_O_2_	220	66.582
9,10-anthracenedione, 2-ethyle-	C_16_H_12_O_2_	236	69.515
Carboxin, carbathiin	C_12_H_13_NO_2_S	235	70.743
9-octadecenoic acid (z)-, methyl ester	C_19_H_36_O_2_	296	93.004
Phenol, 4-(2-aminoproyl)-	C_9_H_13_NO	151	97.475
Hydrocortisone acetate	C_23_H_32_O_6_	404	116.801

## Data Availability

Data of this manuscript are displayed in Figure 1, Figure 2, Figure 3, Figure 4, Figure 5, Figure 6, Figure 7, Figure 8, Figure 9, Figure 10, Figure 11, Figure 12 and Figure 13. The facts and raw data analysed are available from the corresponding author upon request.

## Supplementary Materials

The following are available online at https://www.mdpi.com/article/10.3390/nano11102573/s1, Figure S1: NPs distribution, volume and size for three reading of O-AgNPs sample, Figure S2: NPs distribution, volume and size for three reading of A-AgNPs sample, Figure S3: TEM image presenting shape and size for the distributed O-AgNPs, Figure S4: TEM image presenting shape and size for the distributed A-AgNPs.
