# Supplementary material for: Phytoproduct, Arabic Gum and Opophytum forsskalii Seeds for Bio-Fabrication of Silver Nanoparticles: Antimicrobial and Cytotoxic Capabilities
Source: Nanomaterials (Basel). 2021 Sep 30;11(10):2573. doi: 10.3390/nano11102573 (PMC8538938; doi:10.3390/nano11102573)
Supplement: Supplementary file 1 [file nanomaterials-11-02573-s001.zip › nanomaterials-1385564-supplementary.pdf]

Article

Phytoproduct, Arabic Gum and *Opophytum forsskalii* Seeds for Bio-Fabrication of Silver Nanoparticles: Antimicrobial and Cytotoxic Capabilities

Kawther Aabed * and Afrah E. Mohammed *

Biology Department, College of Science, Princess Nourah Bint Abdulrahman University, Riyadh 84428, Saudi Arabia
* Correspondence: dr.kaabed@gmail.com (K.A.); AFAMohammed@pnu.edu.sa (A.E.M.)

**SOP Name:**	mansettings.nano		
**File Name:**	Example Results.dts	**Dispersant Name:**	Water
**Record Number:**	581	**Dispersant RI:**	1.330
**Material RI:**	1.59	**Viscosity (cP):**	0.8872
**Material Absorbtion:**	0.010	**Measurement Date and Time:**	Thursday, March 23, 2017 8:07:08 ...

**Temperature (°C):**	24.9	**Duration Used (s):**	60
**Count Rate (kcps):**	315.1	**Measurement Position (mm):**	3.00
**Cell Description:**	Disposable micro cuvette (40µl)	**Attenuator:**	11

			**Size (d.nm):**	**% Intensity:**	**St Dev (d.nm):**
**Z-Average (d.nm):**	107.2	**Peak 1:**	121.0	100.0	77.35
**PdI:**	0.264	**Peak 2:**	0.000	0.0	0.000
**Intercept:**	0.862	**Peak 3:**	0.000	0.0	0.000
**Result quality :**	Refer to quality report				

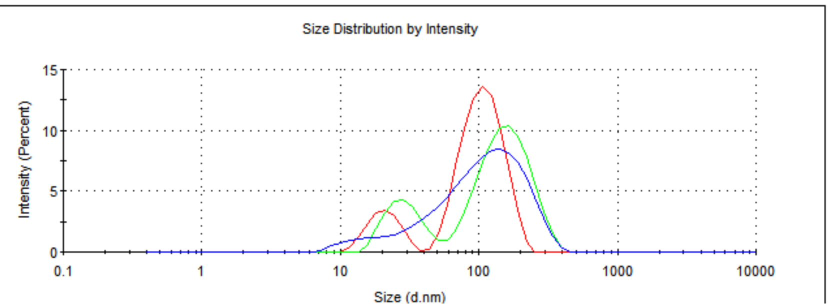

Figure S1. NPs distribution, volume and size for three reading of O-AgNPs sample.

SOP Name:	mansettings.nano		
File Name:	Example Results.dts	Dispersant Name:	Water
Record Number:	584	Dispersant RI:	1.330
Material RI:	1.59	Viscosity (cP):	0.8872
Material Absorbtion:	0.010	Measurement Date and Time:	Thursday, March 23, 2017 8:18:40 ...

Temperature (°C):	25.0	Duration Used (s):	80
Count Rate (kcps):	149.1	Measurement Position (mm):	3.00
Cell Description:	Disposable micro cuvette (40µl)	Attenuator:	7

			Size (d.nm):	% Intensity:	St Dev (d.nm):
Z-Average (d.nm):	75.56	Peak 1:	172.1	86.5	87.65
PdI:	0.680	Peak 2:	12.06	13.1	4.446
Intercept:	0.942	Peak 3:	3.433	0.4	0.5097
Result quality :	Refer to quality report				

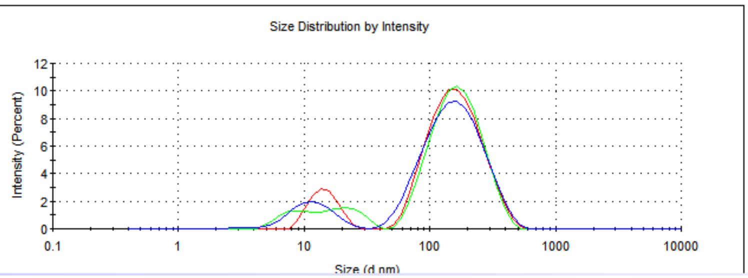

Figure S2. NPs distribution, volume and size for three reading of A-AgNPs sample.

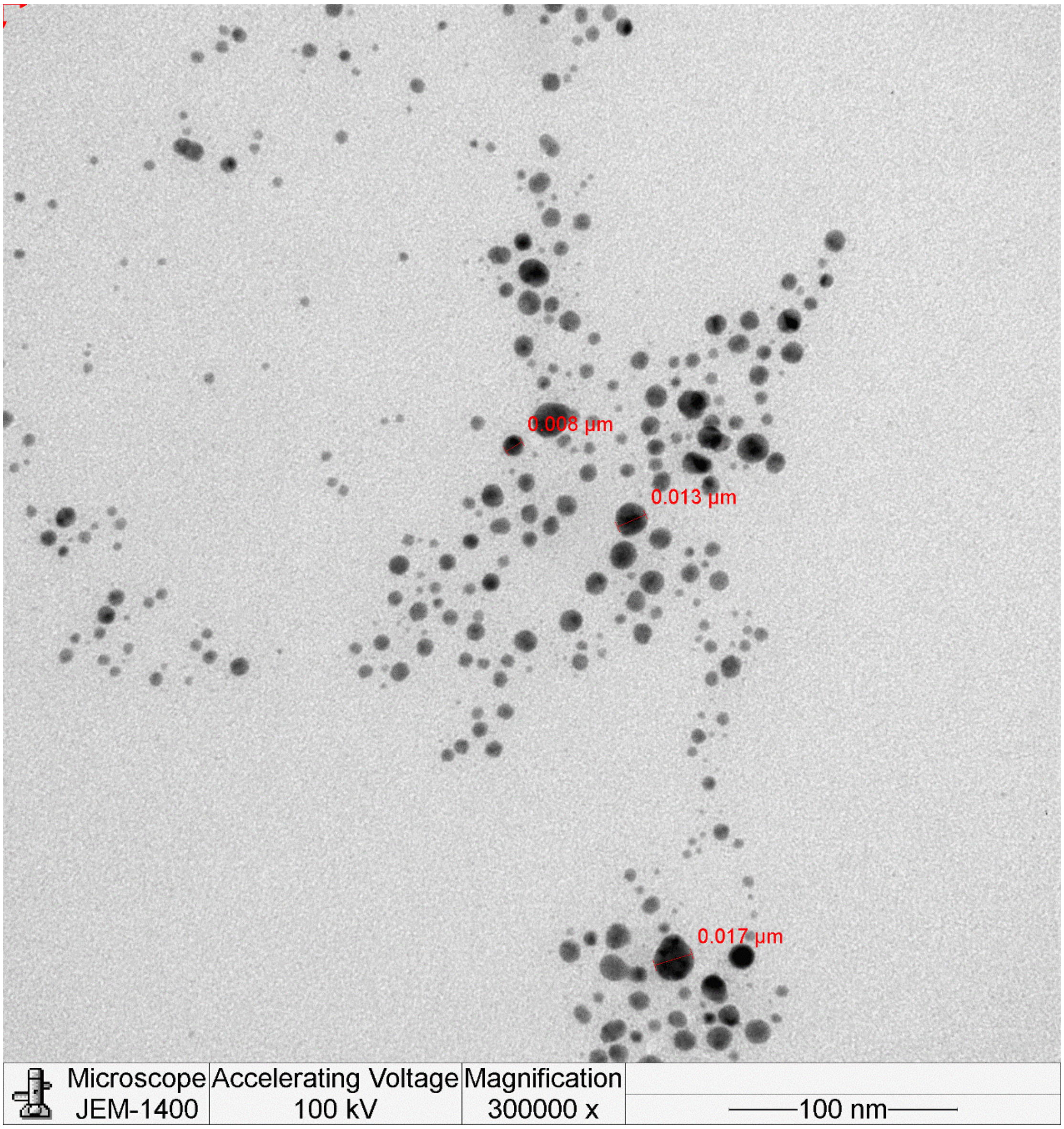

Figure S3. TEM image presenting shape and size for the distributed O-AgNPs

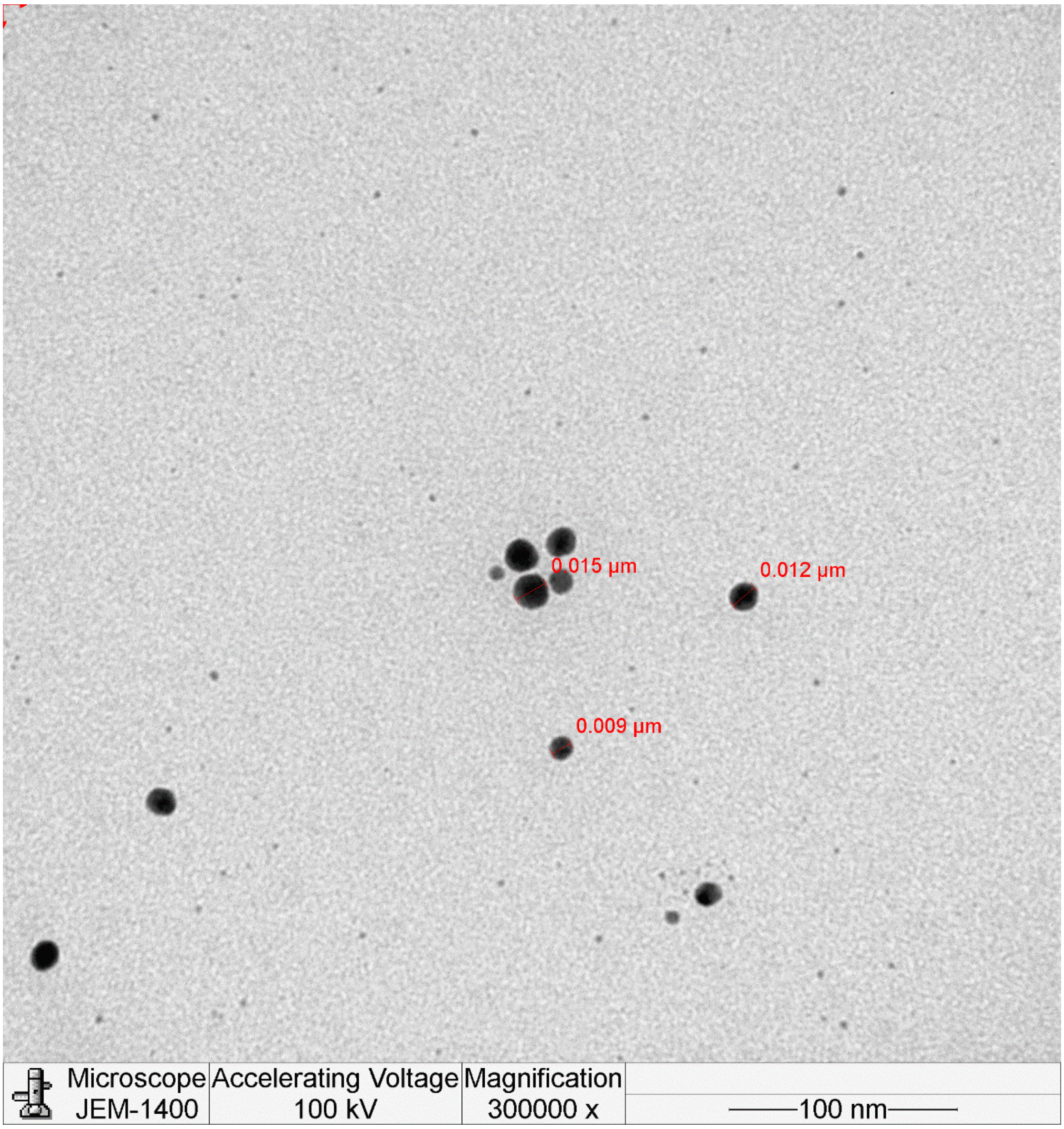

Figure S4. TEM image presenting shape and size for the distributed A-AgNPs
